# Review of Pharmacological and Medicinal Uses of Punica granatum

**DOI:** 10.7759/cureus.71510

**Published:** 2024-10-15

**Authors:** Maureen Mohan, Mohanavarshaa C A, Priya D, Anjana G V

**Affiliations:** 1 Department of Pharmaceutical Chemistry, Sri Ramaswamy Memorial (SRM) College of Pharmacy, Sri Ramaswamy Memorial Institute of Science and Technology (SRMIST), Chennai, IND

**Keywords:** anti-inflammatory, antioxidant, pomegranate, reproductive health, wound-healing properties

## Abstract

Pomegranates (*Punica granatum*) are a fruit renowned for their rich history, diverse uses, and substantial health benefits. Current research on the botanical features, nutritional profile, and medicinal properties of pomegranates is summarized in this study. Botanically, the pomegranate is classified as a deciduous shrub with a unique fruit structure comprising numerous seeds, or arils, embedded in a leathery skin. Nutritionally, pomegranates are a significant source of antioxidants, particularly punicalagin, and anthocyanins, which contribute to their purported health benefits. Emerging evidence suggests that pomegranate consumption may have favorable effects on cardiovascular health, anti-inflammatory responses, and cancer prevention. Clinical studies highlight their potential to enhance blood flow, prevent arterial plaque formation, and reduce the risk of heart disease, heart attacks, and strokes. This review also examines traditional and contemporary uses of pomegranates in medicine and cuisine, highlighting their cultural significance and potential therapeutic applications. Despite promising findings, limitations in current research methodologies and the need for more robust clinical trials are discussed. This review aims to synthesize current research on the botanical characteristics, nutritional profile, and medicinal properties of pomegranates (*Punica granatum*), with a focus on their health benefits and applications in traditional and contemporary contexts. Future research directions are proposed to better understand the mechanisms underlying the health benefits of pomegranates and to optimize their use in preventive and therapeutic contexts.

## Introduction and background

Pomegranates (*Punica granatum* L.; Family: Punicaceae) are red, round, juicy, sweet fruits with arils (edible seeds). Pomegranates are one of the oldest fruit trees known to humans (4000-3000 BCE). They first originated in the Mediterranean region and are widely known for their several medicinal benefits. Including pomegranates in the diet can aid in maintaining blood pressure, cholesterol levels, and blood sugar levels, clearing out digestion problems, sore throats, coughs, heart problems, and urinary infections. Moreover, pomegranates are beneficial in the treatment of various types of cancers such as prostate cancer (PCa), lung cancer, and skin cancer [1]. Clinical research shows that pomegranates have the potential to increase blood flow to the heart, thin the blood, prevent plaque in arteries, and decrease cholesterol, and hence, they are a great help in preventing heart diseases, attacks, and strokes. Pomegranates are well known for their antioxidant and anti-inflammatory effects. They are also a good source of fiber. Pomegranates are mainly composed of flavonoids, ellagitannin, punicalagin, ellagic acid, vitamins, and minerals. Ellagitannin and punicalagin are the principal constituents of pomegranate. Punicalagins are responsible for their anti-inflammatory and antioxidant properties, which have protective aid in heart problems, cancer, urinary functions, brain, and prostate health. The fruits, peels, and seeds of pomegranates have numerous therapeutic effects. Pomegranate juice is used in treating diarrhea and jaundice [2].

The juice of pomegranate's flower is used in treating nose bleeds and gum bleeds, toning the skin, etc. The fruit pulp and the seeds are used for digestive disorders. The seeds of the pomegranate are rich in vitamin C, vitamin K, and folate which helps in reducing the effects of anemia such as fatigue, dizziness, hearing loss, and weakness. Flower buds that are dried and pulverized are used in treating bronchitis. In the food industry, pomegranates are used to manufacture a wide variety of food products such as salad dressings or toppings, candies, jams, juice, and concentrate. The pomegranate waste is used to prepare tooth powders and toothpaste as it is rich in antibacterial and antimicrobial properties which help in treating dental problems such as dental plaques, caries, gingivitis, and mouth ulcers. In India, pomegranate seeds are dried and ground into a powder known as anardana and are used as a spice in chutneys and curries [3]. The taxonomical classification of *Punica granatum* is mentioned in Table 1.

**Table 1:** Taxonomical classification of Punica granatum Source: [1-3].

Kingdom	Plantae
Division	Magnoliophyta
Class	Magnoliopsida
Subclass	Rosidae
Order	Myrtales
Family	Punicaceae
Genus	Punica
Species	P. granatum

## Review

Origin and history, growing conditions, and production 

The pomegranate is also known as the "seeded apple," which is derived from pomum (apple) and granatus (grainy), hence its name. Pomegranates mainly originated in Persia, the center of the Middle Eastern region, then spread to Mediterranean regions, mainly India and China. Its early cultivation was in Ancient Egypt, Greece, Italy, and Iraq. Later, its cultivation was spread to Asian countries, particularly Turkey, Afghanistan, Iran, India, and China. Historical studies show that the pomegranate first appeared around 2000-6000 BP. It was first found growing in the city of Jericho, which is modern-day Israel, during 6000 BP. The cultivation of pomegranates in Central Asia started around 3000 BP. Subsequently, the pomegranate is thought to have spread to subtropical and tropical regions and the rest of the world during the period of the great geographical discoveries around 1600-1700 AD [4].

The pomegranate fruit crops were regularly domesticated, and hence, slight variations arose resulting in larger seeds, larger fruit, color of seeds and fruit, and taste. The usual pomegranate season in the Southern Hemisphere is from March to May and September to February in the Northern Hemisphere. It is mostly drought-tolerant, and it can flourish in dry areas. Hence, it is intensively cultivated and has the best fruit production in the arid regions. India holds the global position of the largest area of pomegranate cultivation and production, whereas Iran is the largest exporter followed by India. Spain holds the first rank in terms of pomegranate productivity followed by Israel. The USA, Turkey, South America, and Australia are among the other countries which are also involved in pomegranate production. In recent years, Chile has also taken the position of the largest pomegranate producer globally [5].

Pharmacological activities for the chemical constituents

Ellagitannins

Ellagitannins are a family of bioactive polyphenols that exist in fruits such as pomegranates, strawberries, almonds, raspberries, and walnuts. They are esters of hexahydroxydiphenic acid and monosaccharide. The pomegranate yields the richest source of ellagitannins. They are mainly recognized for their anticancer properties, especially for PCa. Ellagitannins are hydrolyzable tannins that give ellagic acid on hydrolysis and form urolithins (urolithin A) [6].

Punicalagin

Punicalagin is the largest polyphenol among the ellagitannins, present mostly in the pomegranate peel. It is reported to have more than half of potent antioxidant properties, antiviral, antibacterial, and anti-inflammatory effects. They are reported to have various anticancer properties against several cancer cell lines such as colon cancer, ovarian cancer, PCa, and lung cancer cells by suppressing the proliferation and induction of S phase cell cycle arrest and apoptosis [7].

Gallotannins

One of the other major constituents of the pomegranate is gallotannins. Gallotannins are tannins that are hydrolyzable and can be found in different parts of the pomegranate. Gallotannins have monomeric and dimeric moieties that are derived from aglycone, ferulic acid, p-coumaric acid, and chlorogenic acid [8].

Flavonoids

Pomegranate juice (PJ) is a pool source for flavonoids. There are five subclasses of flavonoids found in PJ which are flavan-3-ols, flavanols, flavanones, flavones, and dihydrochalcones. In addition to flavonoids, even organic acids found in the pomegranate, such as citric acid and L-maleic acid, are being described as one of the most important organic acid constituents in the pomegranate. Furthermore, ascorbic acid, fumaric acid, oxalic acid, succinic acid, and tartaric acid are present in the most identifiable parts of the pomegranate, such as the leaves, fruit peel, and other tissues [9,10].

Anthocyanins

Anthocyanins belong to the flavonoid family, and they are natural hydrosoluble pigments. Anthocyanins are mostly present in the flowers, peels, leaves, and arils. The color of the pomegranate mostly depends on the concentrations of anthocyanins present. Together with hydrolyzable tannins (polyphenols), they produce the overall antioxidant properties. Studies showed that the anthocyanins extracted from the pomegranate arils were used in the preparation of topical creams to evaluate antiaging properties [11]. Six anthocyanins are recurrently occurring in the pomegranate, namely, cyanidin, delphinidin, pelargonidin, petunidin, peonidin, and malvidin. Among all these, cyanidin which contains 3-glucoside is widely distributed and gives the orange-red-purple coloration [12]. Table 2 lists the chemical components of pomegranates.

**Table 2:** Chemical constituents of the pomegranate Source: [6-12].

Constituent	Molecular wt. (g/mol)	Molecular formula	Part
Ellagitannins	992.4	C_44_H_32_O_27_	Seeds, peels
Gallotannins	636.5	C_27_H_24_O_18_	Peels
Punic acid	278.4	C_18_H_30_O_2_	Seed
Linoleic acid	280.4	C_18_H_32_O_2_	Seed oil
Punicalagin	1084.7	C_48_H_28_O_30_	Peel
Olefin	309.2	C_46_H_14_O_2_	Fruit
Hexahydroxy diphenic acid	338.224	C_14_H_10_O_10_	Peel
Flavan-3-ol	226.27	C_15_H_14_O_2_	Fruit, juice
Flavones	222.24	C_15_H_10_O_2_	Fruit
Dihydrochalcones	210.279	C_15_H_14_O	Fruit
Malvidin	331.3	C_17_H_15_O_7_	Fruit, juice
Cyanidin	287.26	C_15_H_11_O_6_	Aril, peel, fruit
Petunidin	317.27	C_16_H_13_O_7_	pericarp, juice
Ascorbic acid	176.12	C_6_H_8_O_6_	Fruit, peel
Fumaric acid	116.07	C_4_H_4_O_4_	Juice
Oxalic acid	90.03	C_2_H_2_O_4_	Fruit, juice
Succinic acid	118.09	C_4_H_6_O_4_	Juice, fruit
Tartaric acid	150.09	C_4_H_6_O_6_	Peel

Medicinal uses of the pomegranate

Polycystic Ovarian Syndrome

Modern research on the pomegranate reveals that pomegranates can balance hormonal levels in the body despite having antioxidant properties. The pomegranate fruit is full of vitamins and minerals that help to regulate the menstrual cycle, help in weight loss, and hold a great benefit to ease other problems associated with polycystic ovary syndrome such as diabetes, heart problems, and infertility. According to the doctrine of signatures, the pomegranate fruit has a likeness to the human ovaries [13].

Cardiovascular Effects

Pomegranates are known for their antihypertensive and cardioprotective effects. The PJ is rich in polyphenols, tannins, and anthocyanins which are potent antioxidants and have anti-atherosclerotic activities [14]. Studies done on hypertensive patients suggest that the PJ greatly reduces the serum angiotensin-converting enzyme activity by 36% after two weeks of pomegranate juice consumption, thereby decreasing the systolic blood pressure [15]. The PJ was found to lower the oxidized lipoprotein of endothelial nitric oxide synthesis in human coronary cells which exerts a beneficial effect on coronary heart disease and the evolution of any clinical vascular complication [16]. 

Diabetes

The PJ has hypoglycemic effects, such as escalated insulin sensitivity, inhibition of alpha-glucosidase, decreased total cholesterol and low-density lipoprotein, and improved blood lipid profiles. The PJ anthocyanins, punicalagins, and ellagic acids are well known to scavenge free radicals and prevent lipid oxidation, since oxidative stress and lipid perfusion are high in diabetic patients [17]. Hence, daily consumption of concentrated PJ is recommended as it increases plasma concentration levels for patients with type 2 diabetes. A study was performed on male albino rats with the administration of aqueous extracts of pomegranate peel at regular intervals which resulted in the reduction in the concentration of glucose content in the serum and lipid peroxidation of the cardiac, hepatic, and renal tissues. The consumption of the PJ shows a significant decrease in macrophage peroxide levels and an increase in the glutathione levels. It was found that pomegranate flowers, when given by oral administration, are said to decrease the content of cardiac triglycerides (TGs), plasma TGs, total cholesterol, and fatty acids [18].

 *Infertility*

Infertility is one of the major issues associated with couples; it is found that around 20% of the population, globally, is affected by infertility. It is found that infertility in males is increased highly around the age of > 35, which further leads to the development of high incidence of systemic diseases, vascular insufficiency, reduced level of sex hormones, disorders in the testis, and mutation and finally affects the oxidative stress.

Over the age of 40, it is found that the male’s semen quality is deteriorating, and the ejaculating volume is reduced.

The pomegranate is determined to be one of the greatest sources to decrease the effect of infertility in males. A study was performed to prove the effect of the pomegranate on male’s infertility. Rats are randomly classified into four categories: Group 1, two-month-old male rats are taken, and saline was given on a regular basis until they turn four months old; Group 2, 10-month-old adult male rats are taken, and saline was given to them for two months until they turn one year old; Group 3, 10-month-old adult male rats are taken, and T-65 was given orally for two consecutive months using oral gavage; and Group 4, rats are taken and given a pomegranate extract at a dose of 250 mg/kg/day using oral gavage for two consecutive months.

It was found that in Group 4, oral administration of the pomegranate increases the fertility of the male rats by 40% [19].

Diarrhea

The pomegranate has been used traditionally in the treatment of gastrointestinal disorders. Aqueous extracts of pomegranate peels gave positive results for alkaloids, flavonoids, and tannins through preliminary phytochemical screening which are prime constituents in treating diarrhea. The aqueous extract of the peel was evaluated using rats. The studies were conducted on the rat isolated ileum, castor oil-induced diarrhea in rats, and gastrointestinal motility in vivo. The results showed that there was a concentration-dependent prohibition of the spontaneous movement of isolated rat ileum, and furthermore, there was a dose-dependent decrease of gastrointestinal transit [20].

Cancer

Breast cancer: Studies show that pomegranate-containing ellagitannin-derived compounds exhibit antiproliferative effects and anti-aromatase activities in breast cancer cells. Polyphenols derived from the fermented PJ and pericarp inhibit 17-beta-hydroxysteroid dehydrogenase. The extract of the pomegranate controls the hormonal level in the blood plasma as it contains estrogenic properties [21]. The unfermented fruit juice of the pomegranate, when consumed, is said to suppress the effect of breast cancer cells. The whole pomegranate seed oil is more chemopreventive for breast cancers [22].

PCa: Studies show that ellagic acid, caffeic acid, luteolin, and punicic acid found in the peel and juice of the pomegranate fruit reduced the invasive potential of PC-3 cells (human PCa cell line). Treatment of the human PCa cells with the pomegranate seed oil, fermented PJ polyphenols, and pericarp polyphenols reduces cell proliferation, increases cells in the G2/M phase, and induces apoptosis [23,24].

Lung cancer: Research gives an account that punicalagin and ellagic acid carry strong antiproliferative properties. Punicalagin decreases the accumulation of oxidative DNA products. The pomegranate peel aqueous extract inhibits neutrophil myeloperoxidase activity [25]. The pomegranate leaf extract reduces the cell proliferation of non-small cell lung carcinoma cell lines [26].

Colon cancer: The pomegranate seed oil consists of 70% conjugated linoleic acid which suppresses colon carcinogenesis [27,28]. The maximum amount of ellagic acid is found in the pomegranate which plays a major role in the anti-inflammatory treatment of lacerative colitis in the development of colon cancer [29].

Skin cancer: The pomegranate fruit extract shows a wide range of activity in inducing ultraviolet B (UVB)-induced phosphorylation of the mitogen-activated protein kinase in epidermal keratinocytes. The occurrence of pretreatment of epidermal keratinocytes with the pomegranate fruit extract results in the inhibition of UVB-induced phosphorylation [30].

Wound-Healing Properties

Wound healing is a complex process. The pomegranate gets involved in the process through various biochemical pathways. The juice of the pomegranate and other extracts of its parts, its phytochemicals, accelerates the wound-healing process, manages pain, decreases the healing period, improves epithelialization, enhances protein and DNA, increases wound contraction, and stimulates collagen and fibroblast production. The pomegranate is also involved in the elimination of microbes that lead to infections. It is rich in polyphenols which are responsible for its anti-inflammatory, antimicrobial, and antioxidant effects; it is used in various systems of medicines and cultures. For example, in the Chinese system and Ayurvedic system of medicine, it is used as an antiparasitic and anti-inflammatory agent and therefore used in wound-healing and ulcer treatments. In ancient Greece, the pomegranate has been used for treating mouth ulcers. In Rome, it is believed that the juice extract is used in the treatment of diarrhea and tapeworms. In the Iranian system of medicine, the pomegranate extracts treat wounds, cuts, and edemas. Hence, the pomegranate extracts greatly help in healing ulcers by reducing the pathogenic count. The pomegranate is also known for its excellent anti-biofilm activities.

A study has been performed to evaluate the wound-healing efficacy of the pomegranate extract for second-degree burns in Wistar rats and is compared to 1% silver sulfadiazine. After 21 days of treatment, the wounds in the pomegranate group were much smaller than in the silver sulfadiazine group.

In another study, creams containing pomegranate flower extract of 5% and 10% were administered to Wistar rats with thermal degree burns and compared with 1% silver sulfadiazine. The creams are directed once every day up to complete wound healing. Results showed that there was a decrease in the average size of the wound in the pomegranate group than in the silver sulfadiazine group from the 15th day of the treatment [31].

## Conclusions

The pomegranate has demonstrated remarkable potential in various aspects of health and wellness. Through its rich phytochemical content, it exhibits antioxidant, anti-inflammatory, antibacterial, antiviral, and antimicrobial effects. Its consumption has shown positive effects on cardiovascular health, cognitive function, and gut microbiota. Nowadays, the pomegranate is even used in the healthcare industries, food industries, and pharmaceutical industries. As a versatile fruit, its seed, juice, and peels also have been utilized in culinary traditions, religious rituals, and natural dyes. This deep-rooted symbolism adds an extra layer of significance to its modern-day applications. As research advances, a comprehensive understanding of pomegranates' bioactive compounds and their interactions will undoubtedly contribute to their integration into personalized nutrition and healthcare products.
